# Machine Learning for Shape Memory Graphene Nanoribbons and Applications in Biomedical Engineering

**DOI:** 10.3390/bioengineering9030090

**Published:** 2022-02-23

**Authors:** Carlos León, Roderick Melnik

**Affiliations:** 1 M3AI Laboratory, MS2Discovery Interdisciplinary Research Institute, Wilfrid Laurier University, Waterloo, ON N2L 3C5, Canada; cleonchinchay@wlu.ca; 2 BCAM—Basque Centre for Applied Mathematics, 48009 Bilbao, Spain

**Keywords:** shape memory effects, DFT calculations, physics-based multiscale modelling, data-driven dynamic environments, knowledge engineering and machine learning, critical size of nanostructures, first-principles studies, biomedical applications, moment tensor potentials, phase transformations, physics-informed machine learning

## Abstract

Shape memory materials have been playing an important role in a wide range of bioengineering applications. At the same time, recent developments of graphene-based nanostructures, such as nanoribbons, have demonstrated that, due to the unique properties of graphene, they can manifest superior electronic, thermal, mechanical, and optical characteristics ideally suited for their potential usage for the next generation of diagnostic devices, drug delivery systems, and other biomedical applications. One of the most intriguing parts of these new developments lies in the fact that certain types of such graphene nanoribbons can exhibit shape memory effects. In this paper, we apply machine learning tools to build an interatomic potential from DFT calculations for highly ordered graphene oxide nanoribbons, a material that had demonstrated shape memory effects with a recovery strain up to 14.5% for 2D layers. The graphene oxide layer can shrink to a metastable phase with lower constant lattice through the application of an electric field, and returns to the initial phase through an external mechanical force. The deformation leads to an electronic rearrangement and induces magnetization around the oxygen atoms. DFT calculations show no magnetization for sufficiently narrow nanoribbons, while the machine learning model can predict the suppression of the metastable phase for the same narrower nanoribbons. We can improve the prediction accuracy by analyzing only the evolution of the metastable phase, where no magnetization is found according to DFT calculations. The model developed here allows also us to study the evolution of the phases for wider nanoribbons, that would be computationally inaccessible through a pure DFT approach. Moreover, we extend our analysis to realistic systems that include vacancies and boron or nitrogen impurities at the oxygen atomic positions. Finally, we provide a brief overview of the current and potential applications of the materials exhibiting shape memory effects in bioengineering and biomedical fields, focusing on data-driven approaches with machine learning interatomic potentials.

## 1. Introduction

Materials with shape memory effects have revolutionized the fields of bioengineering and biomedicine. Some prominent examples of their applications in these fields include sensors and actuators, medical implants, coronary stents, organ frame retractors, and artificial muscles, to name just a few. Shape memory materials (SMMs), a subgroup of intelligent materials, have taken their prominent place in these fields due to their ability not only to sense environmental changes, such as temperature, forces, electromagnetic fields, solvents, and humidity, but also to respond to such changes, adjusting their parameters in order to return to their original state [1]. The role of such parameters can be taken by their shape, position, strain, etc. Therefore, it should not come as a surprise that SMM applications also cover many other areas, well beyond those already mentioned, and may also include deployable components of complex systems, energy conversion, and energy harvesting. Today, such areas as orthopedics and orthodontics, with a wide range SMM-based biomedical devices, are simply unimaginable without an extensive usage of these materials.

Concurrently with the continuing developments in conventional shape-memory materials, more recent research on advanced biomedical applications has also been focusing on graphene-based nanostructures, and in particular on graphene nanoribbons (GNRs, [2]). The latter structures are known for their superior properties, including large surface area, enhanced mechanical strength, and improved electro-conductivity, which make them a major candidate for many applications in biomedicine and bioengineering, including biosensing and diagnostics. GNRs can be used for fast DNA sequencing and can also make good memories, as has been known for quite some time [3,4]. The scope of applications of graphene-based materials, also termed as “smart”, has grown significantly over recent years [5,6]. With the rise of data-driven modeling [7,8,9], machine learning tools and associated methodologies of computational statistics become increasingly important for further progress in these fields [10].

The search for materials that can lead to the fabrication of devices with low-energy consumption has attracted the scientific community to the 2D materials realm. Graphene promises not only that, but also a myriad of exotic properties. Its derivatives can further improve certain desired properties. Graphene oxide (GO) is among these derivative systems that can modulate or enhance the thermomechanical and energy storage properties, due to the presence of oxygen functional groups attached to the layer. Compared to the pristine graphene, GO is cheaper to produce [11] and easy to deposit on a variety of substrates, making it suitable for flexible electronics and bioengineering applications [12,13,14]. GO can be synthesized by either bottom-up or top-down techniques including the Staudenmaier, Brodie, Offeman, Hummer methods [15], and environmentally friendly modifications of them to eliminate the emission of toxic gases [11]. The presence of the epoxide groups alters the electronic band structure and density of states near the Fermi level [16]. As a result, a non-zero bandgap is generated which can be modulated by rearranging the distribution and concentration of oxygen atoms in the surface. The bandgap can be used to take advantage of GO as a luminescent material to be used for biological imaging [17,18]. Epoxide groups can also be used to trap lithium atoms in regions with poor electronic density [19], allowing the use of GO in Li storage devices. GO has also found potential applications for hydrogen storage [20,21] and water purification [22,23].

On the other hand, GO nanostructures can be employed as nanofillers to strengthen mechanical properties of polymers [24] and can be used to fabricate artificial tough nacre of interest in aerospace applications [25,26]. Additionally, GO can endow with shape memory behavior to nanocomposites [27,28] for bone repair with minimal invasive surgery [29], and electrical actuators with low power consumption [30], essential for many applications in bioengineering. Recently, first-principle calculations have shown that GO with highly ordered epoxy groups can experience shape memory effect on its own without the presence of a polymer matrix [31,32] and can experience recoverable strain rates up to 14%. Applications for such shape memory nanomaterials include resonators, artificial muscles, and molecular robots [33], among many others. The carbon–oxygen–carbon interfaces in the GO layer induce the presence of an additional stable phase. The two phases, located at different lattice constant values, are separated by about ∼100 meV. The system can be deformed from one phase into the other one by application of an external force or an electric field [31].

In this work, we use machine learning (ML) to study GO nanostructures, not only to reduce the computational cost involved in the estimation of the two stable phases, but also to analyze the response of GO nanoribbons subject to deformations, and the presence of vacancies and impurities. This work is an extension of [34]. Here, we show different approaches used to improve the model prediction, the approximate critical nanoribbon size for which the shape memory effect is suppressed, and its validation with actual DFT results.

Finally, the machine learning potential is used for a systematic study in which a more realistic distribution of oxygen positions covers the GO surface instead of a highly ordered distribution, the effect of vacancies, and impurities such as boron and nitrogen atoms.

The machine learning technique developed in this paper is part of the group of methodologies known as Machine Learning Interatomic Potential (MLIP). They have been actively developed over the recent years because by controlling the degree of freedoms, the MLIP functional forms allow us to consider different environments and to use more elaborate descriptors of local atomic environments. In bridging the gap between costly DFT calculations and less accurate classical potential methods, we develop a data-driven method known as Moment Tensor Potentials (MTP) for shape memory graphene nanoribbons. As such, the MTP is a powerful multipurpose tool that can be used: for training (as in linear regression, it finds the coefficients that minimizes a function that depends on the energy, forces, and stress values found in static DFT results), for structural optimization (instead of relaxing the structure via DFT), as well as for energy prediction only. One of our main motivations to choose the MTP method for ML in this paper was due to the fact it works directly with atomic environments and already makes use of physical restrictions. While for other ML techniques, it would have been necessary to choose first an appropriate representation (see, e.g., [34] and Section 4 for further details), the methodology developed here in Section 2 and Section 3 allows a robust trade-off between accuracy and computational cost. Not only we have demonstrated the proof-of-concept as well as the efficiency of the developed MTP algorithm on a class of important problems considered here, we also have provided a series of reproducible illustrative examples. Such examples have been given for a significant class of innovative nanostructures that are poised to be vital in many bioengineering and other applications.

## 2. Methods

Given available options to tune their electronic, mechanical, and optical properties, graphene oxides (as well as reduced graphene oxides) have been playing an important role in many applications of graphene and its derivatives [35]. In what follows, we will describe our methodology to analyze GO layers first, moving to its generalization for GO nanoribbons as the next step.

**Figure 1:**
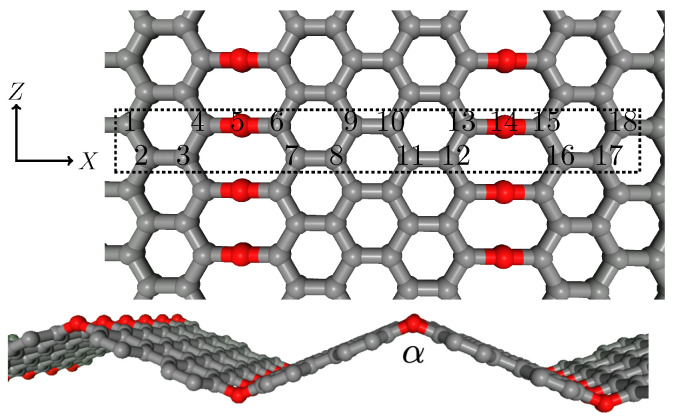
(**Top**) GO structure before structural optimization. The unit cell is shown in between dashed lines. The unit cell’s length along the *X*-direction defines the lattice constant, $a_{\mathrm{lat}}$. (**Bottom**) Side view of the optimized GO structure using DFT. Gray (red) balls represent carbon (oxygen) atoms.

From the different arrangements in which a highly oxygen-ordered configuration can be found in a GO layer, we focus our attention on the $C_{8}O$ structure defined in [31] since it exhibits shape memory effect and the two different phases in which it can be energetically stable. The unit cell is depicted in Figure 1. The periodicity of the unit cell along the *X* and *Z* directions defines a 2D sheet. From the figure, it can be noticed that the oxygen epoxy groups define graphene stripes of zigzag interfaces. The electronic re-arrangement around the zigzag interfaces bends the 2D layer at angle α, as shown by the optimized DFT structure also represented in Figure 1. According to [31], the GO sheet presents two phases around α=104° and 133° [31,32], corresponding to lattice constants of $a_{\mathrm{lat}}∼16\;Å$ and 18.5Å. The two phases are separated by ∼100meV. First-principles DFT calculations will be used to validate literature results.

Specifically, we perform spin-polarized DFT calculations to relax the atomic positions at several fixed lattice constants to build the energy vs. lattice constant curve. All of our DFT calculations were performed by using the Quantum ESPRESSO simulation package v.6.8 (see, e.g., [36]), plane-wave basis sets, and ultrasoft pseudopotentials [37], while employing the gradient approximation with the PBE exchange-correlation functional [38]. Our tests of convergence showed optimal values for a wavefunction energy cutoff of 60Ry and a 4×1×4 Monkhorst-Pack k-point grid (periodicity is along the *X* and *Z* directions). Here we used an interlayer separation of 18Å to ensure a minimum distance of ∼12Å between atoms, even with a bent structure for the range of lattice constant values considered (from $a_{\mathrm{lat}}=14\;Å$ to $a_{\mathrm{lat}}=19\;Å$).

### 2.1. Moment Tensor Potentials (MTP)

After the validation, we have employed ML approaches that can mimic the DFT results and allows us to predict the behavior of nanoribbons with very big super unit cells, multiples of the unit cell shown in Figure 1 with periodicity only along the *X*-direction. We use a physics-based ML model designed for materials, coded in the MLIP (Machine Learning Interatomic Potential) package [39] that we use to build an interatomic potential for the GO system.

The MLIP code is based on moment tensor potentials (MTP) [39,40]. By utilizing active learning, the construction procedure leads to an automatic sampling of configurations for the training set in an efficient manner, as well as indicates a way to expand the training set amenable to the concurrent analysis of the prediction errors. In this machine learning approach, the quantum mechanical energy of a structure, $E_{\mathrm{QM}}$, is approximated as a sum of interatomic potentials, *V*,

(1) $$E_{\mathrm{QM}}\approx \sum_{i}V\left(n_{i}\right),$$

where $n_{i}$ represents the neighborhood of the *i*-th atom, given by a collection of atomic positions and species of each neighbor atom up to cutoff radius. *V* is expressed as an expansion of polynomials

(2) $$V\left(n_{i}\right)=\sum_{i=1}^{N}c_{\alpha }B_{\alpha }\left(n_{i}\right),$$

where the expansion in the polynomial $B_{\alpha }$ and its construction ensures *V* to be invariant to structure’s rotation and permutations of the same-species elements. $B_{\alpha }$ is build in terms of the moment tensor potentials $M_{\mu ,v}$ defined as

(3) $$M_{\mu ,v}\left(n_{i}\right)=\sum_{j}{\left|r_{i}\right|}^{2\mu }r_{i}^{⊗v},$$

where $r_{i}^{⊗v}=r_{i}⊗\ldots ⊗r_{i}$ indicates the Kronecker product of *v* copies of the $r_{i}$.

The model is trained by finding the coefficients $c_{\alpha }$ that best fit not only the quantum mechanical energy of the system in expression (1), but also the forces on each atom (derivative of (1) with respect to atomic positions), and stress values (proportional to the derivative of (1) with respect to lattice constants), values that were found in static DFT results.

The model built through the learning of energy, forces, and stress values can be used to relax the atomic positions. In the relaxation process, the different configurations generated are graded according to geometric considerations. If a configuration is found to be an extrapolation from the training set and its grade is higher than a threshold grade value (“active learning”), a static DFT calculation is required on the new configuration to obtain its energy, forces, and stress.

### 2.2. ML Implementation for a GO Layer

We generate an initial set (IS) of 41 artificial structures as shown in Figure 1 with lattice constant values over the range from 14 to 20Å to analyze the GO energy dependence on the structure length along the *X*-direction and learn from the associated stress. Atomic equilibrium positions were approximated to a pristine graphene nanoribbon with a carbon-carbon distance of 1.42Å.

Given the IS, the relaxed structures have been found by MTP and compared to DFT calculations. In the process of building the interatomic potential, a training set (TS) is generated, from which static DFT calculations are performed. Now, the interatomic potential is created. However, it is possible to build a new potential with higher accuracy prediction by choosing new configurations closer to the ground state, although the new potential will have an energy prediction valid for a window of energies restricted to the neighborhood above the ground state.

### 2.3. ML Implementation for GO Nanoribbons

Finally, by using the ML interatomic potential methodology, we extend our study to a class of graphene nanoribbons. Graphene nanoribbons have been studied extensively in the context of various applications (e.g., [41,42,43,44,45,46] and references therein), including biomedical [47,48]. As we already mentioned in Section 1, graphene itself is considered to be a smart material [5], and the range of graphene-based smart materials and their applications continues to grow [6]. Our main attention in this paper has been given to the class of armchair GO nanoribbons (AGONRs).

The training set is composed of DFT optimized structures with different widths. The provided atomic environments will be used to train an MTP model, which will be used as an interpolation tool to estimate the energy dependence over the lattice constant. This approach will let us study the effect of confinement on the shape memory behavior of a GO sheet, and the evolution of the two phases at finite nanoribbons widths.

The ML interatomic potential allows us to investigate GO nanoribbons of large widths compared to what is accessible through DFT calculations. Oxygen vacancies and lower ordered epoxy groups are considered as parts of realistic configurations that can be found in GO. We also analyze the effect of boron and nitrogen atoms replacing oxygen atoms.

## 3. Results and Discussion

**Figure 2:**
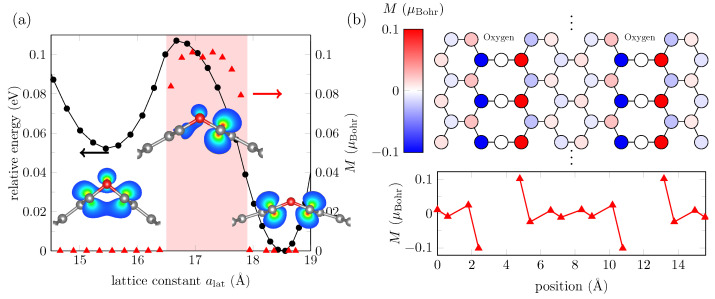
(**a**) Energy (black marks) and maximum magnetization (red marks) found in optimized GO nanoribbons of different widths, according to DFT calculations. The red shadowed area indicates non-zero magnetization for structures in the corresponding lattice constant range. The insets show partial charge densities around the oxygen atoms (red balls) and carbon atoms (gray balls) for three $a_{\mathrm{lat}}$ values: 15.5Å, 17Å, and 18.5Å. (**b**) Magnetization at each atomic site in the unit cell of a GO layer of $a_{\mathrm{lat}}=17\;Å$.

DFT validation is shown in Figure 2a. The 2D system exhibits a stable phase at ∼18.5Å and a metastable phase at ∼15.5Å, separated by 60meV. The symmetry evolution of the electronic wavefunction can be noticed in the inset of the same figure. The electronic distribution around the oxygen atoms is rearranged as the system is shrunk into an anti-bonding-like configuration, with higher energy, and then again into a bonding-like configuration, for ∼17Å and ∼15.5Å, respectively. The electronic rearrangement around the bendings in the red shadowed area in Figure 2a behaves as quantum wells, effectively isolating the zigzag graphene nanoribbons (ZGNRs) between the rows of oxygen atoms. The electronic repulsion at carbon atoms in isolated ZGNRs leads to ground state magnetic solutions. The magnetic distribution in ZGNRs is similar to Figure 2b, where magnetization reaches a maximum value at the edges. The application of an electric field has a parallel component to each ZGNRs plane, and it leads to a rearrangement of charges across the zigzag interfaces that could break the isolation of the ZGNRs, and hence lowering down the energy barrier between the phases. As shown in [31], the application of the right electric field intensity destroys the energy barrier between the two phases and generates instead a new global energy minimum. Therefore, the application of an electric field shrinks the system into a new lattice constant. In what follows, we will use ML to learn from this structure with no electric field applied.

### 3.1. ML Results for a GO Layer

**Figure 3:**
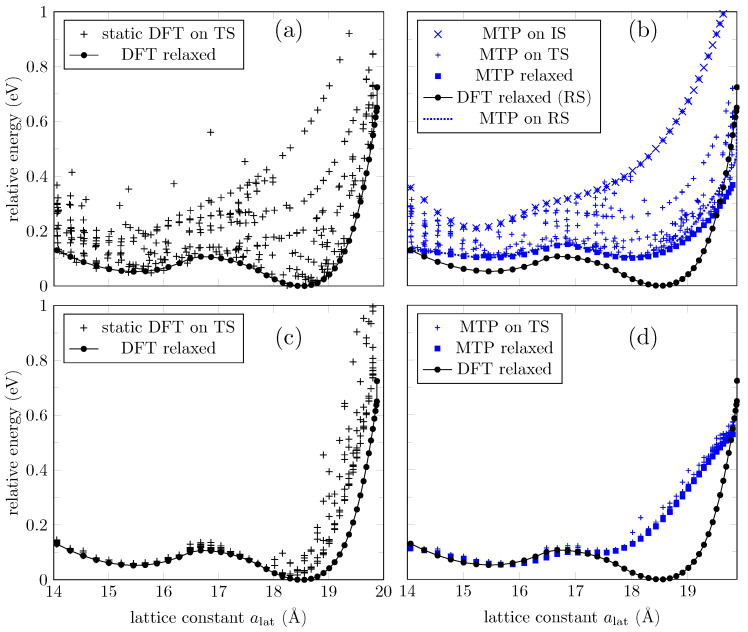
Energy of a GO layer via DFT and MTP. (**a**) Energy distribution of the training set (TS) compared to the ground state energies according to DFT calculations (circle marks). (**b**) Energy distribution of the input set (IS) and TS according to the ML potential. The IS has been relaxed using the ML potential (blue circle marks) and DFT (black circle marks). (**c**) Same as (**a**), but with an improved IS. (**d**) Energy distribution of the TS according to an improved ML potential.

**Figure 4:**
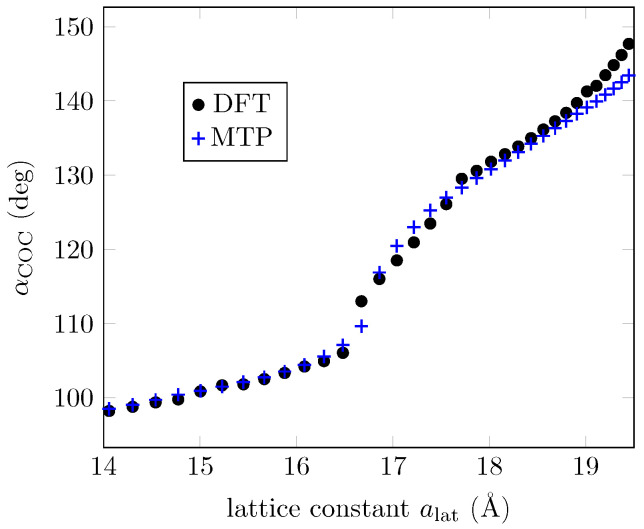
DFT-optimized structures compared to MTP predictions: C-O-C angles.

The 41 structures in the IS were used to start the iterative process of selection, training, and relaxation. Convergence tolerance is achieved, and the RMSE of the trained potential is 75meV (or 4meV per atom). Overall, 395 configurations are generated as the TS, and static DFT results are used to feed the training process. The TS spans over 1eV above the ground state configurations, as shown in Figure 3a. We use the trained interatomic potential to reproduce the DFT energies in the TS and to relax the configurations in the IS. Figure 3b gives a better idea of the extent of the accuracy of the trained potential. While it can predict accurately enough for configurations with higher energies, it fails for configurations around the second phase (∼18.5Å) as the resulting value exceeds the RMSE of the trained potential mentioned earlier in this section. This happens only for the energies around this second phase. Two quantities, the energy and C-O-C angles, obtained with the MTP and DFT for the two minima, are provided in Table 1. It should be emphasized that these data may not be considered as a basic attribute characterizing the model in this case. What is important is that the model can be used to filter structures in the search for candidates to be the ground state, so it is where we can find essential applicability of the ML model. For completeness, in Figure 4 we also present the C-O-C-angles evolution according to DFT, and according to the ML potential used for the 3d graph. Despite the limitations in predicting the energies mentioned above, one can see that the C-O-C-angles of the ground state configurations calculated via MTP are superposed with the DFT results.

Next, we extract the atomic positions of DFT relaxed configurations (“RS”) to verify if the ML energy prediction agrees with DFT. The ML potential agrees that those are the ground state configurations (“MTP relaxed” and “MTP on RS” curves coincide in Figure 3b), but again the energy prediction is higher around the second phase (see “DFT relaxed” curve in Figure 3b). Still, the ML potential can find the two local minima around the two phases.

To increase the accuracy of the ML potential, we now take as IS to be the 41 MTP relaxed configurations in the previous process (configurations for which energies were plotted as the “MTP relaxed” curve in Figure 3b). In this specific case, the new IS already coincides with the DFT relaxed configuration, as discussed above. The iterative process of selection, training, and relaxation is repeated until convergence is achieved. The RMSE is 111meV (or 6meV per atom). Overall, 386 configurations were generated by MTP to train the ML potential. Figure 3c shows the energy distribution of the new TS compared to the ground state energies. We can notice that the TS includes ground state configurations up to ∼18.5Å only. The new ML potential is used to reproduce the energies of configurations in the TS, and the results are shown in Figure 3d. We also employ the ML potential to “relax” the IS (although they already coincide with the DFT relaxed configurations); we expected the energies to coincide with the ground state according to DFT. The results are exhibited as the “MTP relaxed” curve in Figure 3d. The curve partially recovers the DFT ground state energies, but fails to predict the behavior around the second phase. This result is in part due to the lack of the required configurations in the TS around the second phase, and in part due to the long-range interactions introduced by spin-polarized carbon atoms (see Figure 2b) that MTP is not able to learn from. MTP approximates the quantum mechanical energy of the system to a sum of local energies (Equation (1)), and as stated in [40], the assumption could not be valid at all for systems with long-range interactions.

**Figure 5:**
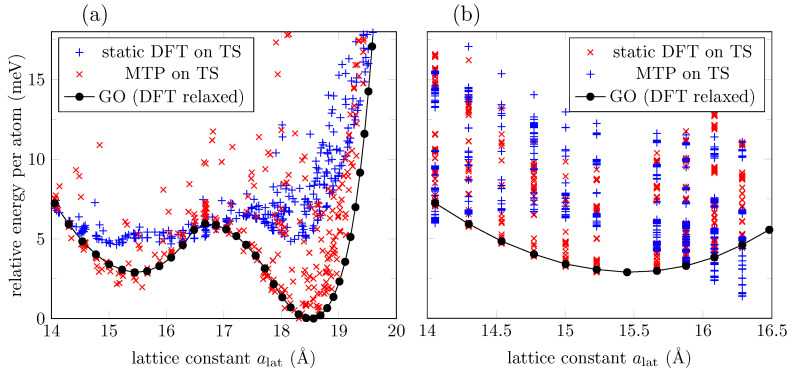
(**a**) Energy distribution of the TS for a model trained using a cutoff of 10Å, according to DFT and MTP. (**b**) Energy distribution of the TS for a model trained on a restricted range of lattice constant values from 14Å to 16.5Å. For comparison, ground state energies, according to DFT calculations, are provided (black marks).

Letting MTP learn from larger atomic neighborhoods to account for long-range interactions might improve accuracy prediction. MTP learns from each atomic neighborhood (Equation (1)) and so far we have worked with a default cutoff value of 5Å, which includes interactions up to the third nearest neighbors in a graphene derivative. However, choosing an ML potential with a larger cutoff of 10Å does not seem enough to improve accuracy prediction at low energies, as shown in Figure 5a.

Magnetic solutions introduce additional complexity that MTP cannot capture to make accurate energy predictions. Hence, we will focus on a lattice constant range for which no magnetic configurations have been found. The ML potentials have shown better accuracy prediction around the first phase, so we now restrict the IS and TS to configurations with $a_{\mathrm{lat}}=14\;Å$ to $a_{\mathrm{lat}}=16.5\;Å$. Results are presented in Figure 5b. However, the generated TS includes more than 1000 configurations, so it is no longer computationally cost-effective to continue trading for a higher accuracy prediction.

### 3.2. ML Results for GO Nanoribbons

Training an ML model for the highly oxygen ordered $C_{8}O$ layer involves the learning of atomic environments of 18 atoms per unit cell. For GO nanoribbons, ML training might result challenging due to the computational cost involved in the DFT calculations of the TS (even though they are static calculations), except for very narrow strips. Therefore, to build the ML model for nanoribbons, we choose relatively narrow nanoribbons that have been DFT relaxed, and restrict our ML model to work with configurations very close to the ground state configurations. In this part, we are not following the selection, relaxation, and training iterations as we did for the 2D GO. Here, MTP is not used to relax structures, instead we use MTP only as an interpolation tool to find the approximate energies of GO nanoribbons. To generate the TS, we combine DFT results for relaxed nanoribbons and the 2D GO, so that MTP can find the interpolated energies for structures all the way from 1D to 2D structures.

**Figure 6:**
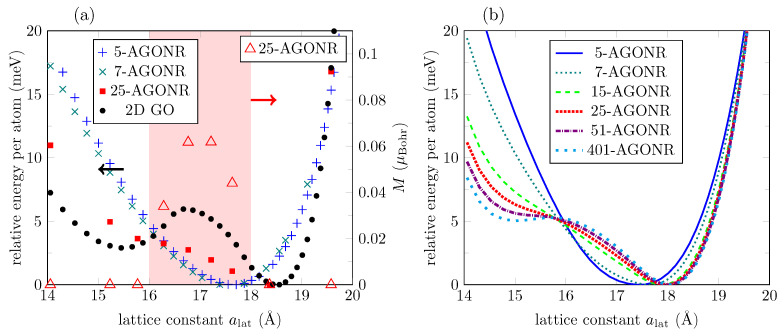
(**a**) Energy evolution of relaxed *m*-AGONRs with m=5,7,25 armchair lines, according to DFT calculations. For comparison, ground state energies of the GO layer are included (black circle marks). The red shadowed area indicates non-zero magnetization for a nanoribbon with m=25 in the corresponding lattice constant range. (**b**) Energy evolution for GO nanoribbons with different widths according to the ML potential.

Figure 6a displays the evolution of DFT relaxed nanoribbons of different widths compared to the GO layer. While the GO layer has a global minimum at ∼18.5Å, narrow nanoribbons have a minimum at ∼17.5Å. However, the global minimum shifts to larger lattice constants for wider nanoribbons, as revealed by the 25-AGONR’s evolution. Additionally, narrow nanoribbons do not present ground state magnetic solutions. This scenario changes for 25-AGONR, in which maximum magnetization is no longer zero, but it is at half range to the maximum magnetization found for the 2D GO (compare triangle marks in Figure 6a and Figure 2a). Finally, Figure 6a shows that while 5- and 7-AGONRs evolve with a very well defined convexity, the 25-AGONR presents a noticeable change in the gradient at around ∼15.5Å, i.e., the formation of an additional phase begins to take place, although there is no local energy minimum defined yet.

As explained above, we combine DFT results for nanoribbons and the 2D layer to construct the TSs, which are used to train ML potentials. Several TSs can be built: 3-AGONR/GO, 5-AGONR/GO, 7-AGONR/GO, and combinations between them. The ML model with TS as 3-AGONR/GO can describe well the convergence to the 2D system, but overestimate the energies for narrow nanoribbons, possibly due to an overestimation of the edge to edge interaction, which should decrease rapidly for nanoribbons with higher widths. The potential trained with TS as 5-AGONR/GO can reproduce better DFT results for narrow nanoribbons, but fails to reproduce the formation of the two phases at the 2D limit, possibly due to the lack of enough oxygen-oxygen interaction data (5-AGONR has only 2 Oxygen atoms at each bending). Figure 6b presents results for the best ML potential we found, in terms of convergence to the 2D limit, the evolution of the two phases for nanoribbons with higher widths, and the low energy prediction error for narrow nanoribbons. The ML potential was trained with TS as 7-AGONR/GO. It predicts the change in the gradient for the 25-AGONR energy curve (25-AGONR DFT results are not contained in the TS), and it can even describe the shift of the global minimum for wider nanoribbons.

Furthermore, Figure 6b tells us that the stable phase is rapidly created when increasing the nanoribbon width, while the metastable phase (at $a_{\mathrm{lat}}$ ∼ 15Å) gradually appears at a slow rate. Therefore, the metastable phase is associated with the formation of new states due to the oxygen–oxygen interactions in the same row that strengthen as the width increases, and converges at the 2D limit. According to MTP, the first signatures of the metastable phase are predicted to appear around nanoribbons with 25 armchair lines (3nm wide), results that are confirmed by DFT calculations. For 51-AGONR, the metastable phase is still evolving. For 401-AGONR, around ∼50nm width, a clear local minimum is obtained. This critical value seems reasonable when compared to the critical size of 60nm for FePd nanostructures, either with experimental results [49] or via a phase-field model [50]. Here, we have used a different approach on a different system to find a critical width in which the effect of confinement suppresses the shape memory behavior in GO nanoribbons.

### 3.3. Effect of Oxygen Defects in GO Nanoribbons

**Figure 7:**
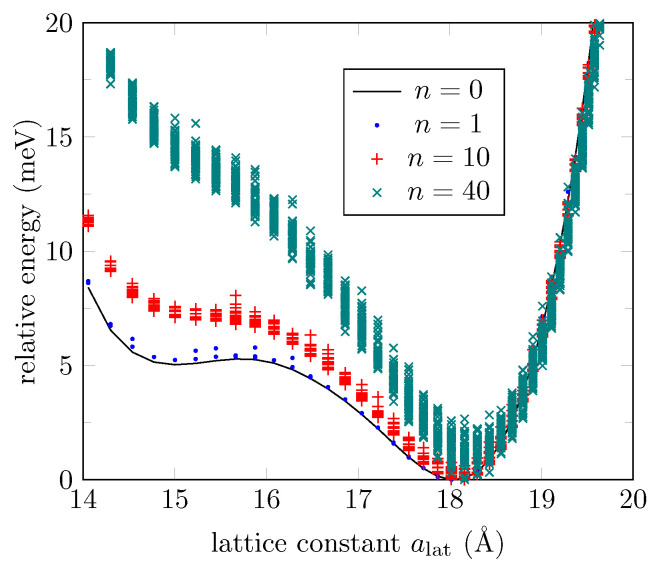
Energy distribution, according to the ML potential, of a pristine 401-AGONR (n=0), and in the presence of n=1,10,40 vacancies out of 400 oxygen atoms at random positions. Fifty samples are collected for each lattice constant and for each *n*.

The robustness of the second phase in the presence of imperfections can now be evaluated through the same ML potential built above (using a TS as 7-AGONR/GO). We consider the case in which the bendings of the GO nanoribbon are partially filled with oxygen atoms. Figure 7 shows the distribution of energies for a 401-AGONR at 0.25%, 2.5%, and 10% of vacancy concentrations (1, 10, and 40 out 400 oxygen atoms, respectively). For each lattice constant value, 50 samples are generated by randomly removing oxygen atoms over the bendings, so the energies are spread over a range that could remove local minima. As expected, low vacancy concentrations lead to configuration energies close to the ground state. In contrast, the second phase is destroyed for vacancy concentrations around 10%.

**Figure 8:**
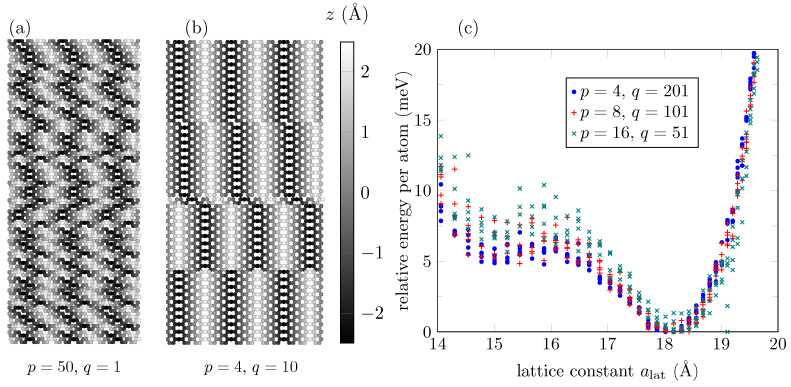
(**a**) Unit cell of a 50-AGONR with no ordered oxygen atoms over the surface (q=1). (**b**) Unit cell of a 40-AGONR with partially ordered oxygen atoms, with the order covering a length of q=10 armchair lines. Periodicity is along the horizontal direction (*X*-axis). Atoms are represented as balls in gray color levels to indicate their atomic height positions over the XY-plane. (**c**) Energy distribution of a 800-AGONR with partially ordered oxygen atoms at different levels, for q=201 (highly ordered), 101, and 51.

So far, we have studied GO with highly ordered oxygen rows. We now address the case of a more realistic configuration in which oxygen atoms adhere to the GO nanoribbon surface at random positions, but are still restricted to the $C_{8}O$ stoichiometry, as shown in Figure 8a. Yet, the preference for the formation of ordered oxygen rows can be modeled by keeping the same oxygen positions over *q* armchair lines, and then by choosing randomly a different oxygen position for the next *q* armchair lines. A balance between totally random oxygen positions and ordered rows is depicted in Figure 8b. Defects between the ordered interfaces, new oxygen–carbon atom interactions, and other long-range interactions could affect now the nanoribbon energy, as shown in Figure 8c for q=201, in which the second phase still can be found (in the plot, 5 energy samples per lattice constant value were collected using configurations with ∼10,800 atoms per unit cell). The second phase is not totally destroyed even for higher frequent change in the orderings (p=8, and p=10 in the figure), provided that the ordered strips formed between line defects are wide enough to exhibit the two phases if they were isolated from each other (above 51 armchair lines wide).

### 3.4. Effect of Boron and Nitrogen Substitutions

**Figure 9:**
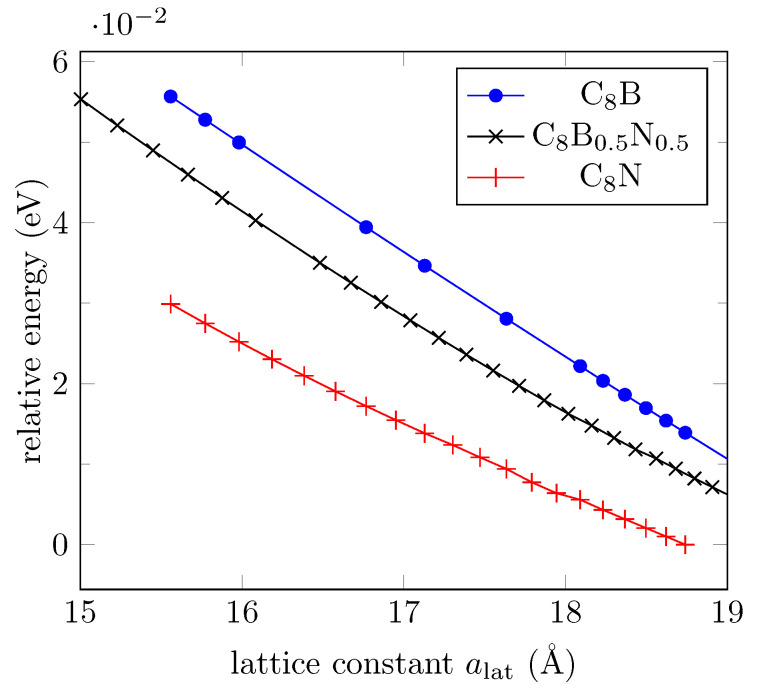
Energy evolution of relaxed GO layers when all oxygen atoms are replaced by boron (blue marks) or nitrogen (red marks) atoms or a pair B-N (black marks), according to DFT calculations.

Other realistic configurations include substitutions of oxygen atoms by impurities. We choose boron and nitrogen atoms as substitutes since the valence electrons of B and N together equal to those of a pair of C atoms. It is known that substitution of carbon by boron atoms in an armchair graphene nanoribbon (AGNR) breaks the conjugated electron system, creating a reflective barrier for the π-electrons, and can induce magnetized edge states [51]. The ordered oxygen atoms in the disposition shown in Figure 1 also define localized states for which a finite magnetization has been found for an interval of lattice constant values. However, oxygen substitutions by boron or nitrogen atoms destroy the two-phase feature, and hence its shape memory behavior. We include here DFT results for structural optimization for oxygen substitutions either by boron or nitrogen atoms ($C_{8}B$ or $C_{8}N$), or with a boron–nitrogen pair of atoms ($C_{8}B_{0.5}N_{0.5}$). Figure 9 shows the relative energy in each case for different lattice constant values, with bending angles from $100^{\circ }$ to $180^{\circ }$ (flat system). The two-phase feature displayed by $C_{8}O$ is lost.

**Figure 10:**
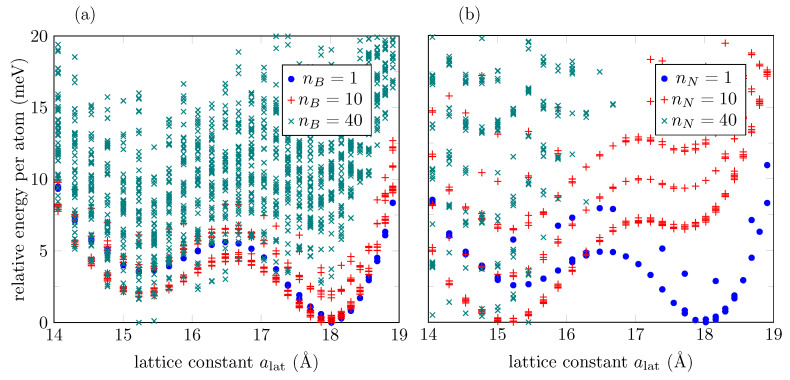
(**a**) Energy distribution, according to the ML potential, for a 401-AGONR where $n_{B}=1,10,40$ oxygen atoms out of 400 are replaced by boron atoms at random positions. (**b**) Same as (**a**) for nitrogen substitutions. Fifty samples are collected for each lattice constant and for each $n_{B}$ or $n_{N}$ values.

We follow a similar procedure to generate configurations with oxygen vacancies in the previous section using the ML potential. Random oxygen atoms were chosen to be replaced either by boron atoms, presented in Figure 10a, or nitrogen atoms, shown in Figure 10b. Low boron substitution concentration (0.25%: $n_{B}=1$ out of 400 oxygen atoms) does not exhibit noticeable change. The two-phase feature is only destroyed for a 10% boron substitution concentration. This result contrasts with nitrogen substitutions, in which the two-phase feature is already affected by a 0.25% concentration, and destroyed for a 2.5% concentration.

## 4. Data-Driven Approaches for Studying Materials with Shape Memory Effects in Biomedical and Other Applications

Our main focus in the previous sections has been on an important emerging class of materials with shape memory effects, specifically graphene-based nanostructures for which we have developed machine learning tools allowing their further advanced studies. Applications of such materials, including shape memory GNRs, are still in their early, but very active development phase [46,52,53]. In the meantime, other classes of materials with shape memory have a well-established range of biomedical and other applications and we believe it is important to provide a brief overview of the situation, also because the type of MLIP techniques we have developed in the previous sections can potentially be useful for more efficient treatments of problems in already existing SMM applications. On the other hand, the ideas we discuss below in the context of SMMs could certainly be useful for multiscale analyses of GNRs after their integration into actual biomedical devices or structures. While the classes of SMMs we discuss in this section are much better developed from a modeling point of view, this cannot be said about shape memory nanomaterials to which we have devoted the main part of this paper. Yet, both require the development of data-driven approaches such as those based on ML and DL. Hence, our brief review of other classes of materials with shape memory effects in the context of biomedical engineering will be followed by a discussion on the most recent developments in such data-driven approaches, paying special attention to the methods preserving physical invariant properties and to MLIP functional forms permitting considerations of different environments and using more elaborate descriptors of local atomic environments.

In designing materials for biology and medicine, materials with shape-memory effects have played a remarkable role [54]. From efficient responses to injuries and delivering drugs to hardening techniques in producing casts on broken bones, and to implants and prostheses, the healthcare system of today relies heavily on SMMs. Many areas, including orthopedic surgery and orthodontics, are simply unimaginable without these materials. Moreover, SMMs continue molding many minimally invasive therapies and related bioengineering technologies. Indeed, these materials not only assist in accelerating wound healing, provide self-expandable vascular stents, and result in high clinical effectiveness, but also aid in developing new biomedical appliances, thanks to their biocompatibility properties. They are a critical component for biotribological systems [55,56] which, in their turn, inspire the development of new technologies in bio-related fields. Furthermore, SMMs for biocompatible microactuators, as biocompatible suture materials for tissues such as tendon [57], also showed excellent cytocompatibility properties for better cell adhesion and morphology in different cell culture systems [58]. SMMs have been vital in various methods of tissue engineering, including distraction osteogenesis [59].

From the modeling point of view, much research in these fields is based on the phase-field theory, where the Landau order parameter framework can be applied in the areas ranging from traditional materials science to the phase transitions in the genome-wide dynamic networks and other complex systems. For example, single-molecule stretching experiments on DNAs, RNAs, and other biological macromolecules opened up the possibility of impressive progress in many fields of life and medical sciences [60], and the methodologies for the analysis in these areas are frequently based on non-convex free energies, which is akin to the analysis of force-induced martensitic phase transformations in SMMs. Since reversibility of structural phase transformations in these materials has profound technological implications, covering many areas including bioengineering, it is important to develop dynamic models of underlying processes [61]. Coupled thermoelastic martensitic phase transformations and microstructure evolution are behind these processes. At the same time, in order to make the dynamic problem tractable in engineering applications, a dimensional reduction of the fully coupled dynamic three-dimensional model for SMMs needs to be carried out, which was for the first time proposed in [62]. The reduced model was approximated by a system of differential-algebraic equations and was applied to the modeling of SMM-based devices such as actuators [63]. This approach allowed us to study both stress- and temperature-induced phase transformations and associated hysteresis phenomena in SMM structures in a unified manner [64] and to extend the developed technique to the Cattaneo–Vernotte law for heat conduction, following principles of extended thermodynamics in the context of SMMs [65]. The problems at hand require the development of multiscale approaches [66] and by now we know that a synergy between multiscale modeling and machine learning can provide a very powerful tool for this research [67]. For example, in [68] polycrystalline structures with microstructure properties have been a subject of studies with machine learning and deep learning approaches, combined with multiscale analysis. The interest in machine learning tools for studies of martensitic phase transformations, typical for SMMs, has been growing significantly over recent years [69], and this direction of research has also included various approaches for developing interatomic potentials [70].

In parallel with the above research, the search for new composition regions of SMMs to achieve improved control of SMM properties has been underway [71]. One of the key candidates for this search has always been polymeric materials that exhibit shape memory properties. They come in a wide range of forms, from those that can be extracted from biological oils [72] to those that use carbon nanotube reinforcement [73], with many other forms of shape memory polymers and their composites currently under investigation [74,75]. Indeed, each class of such smart, stimuli-responsive shape-memory polymers is designed according to the specific requirements [76], e.g., programmed to a specific temporary shape and such that they can recover their original shape upon the application of various stimuli, depending on a specific application (temperature, electric and/or magnetic field, solvents, light irradiation, etc.). In such cases, shape memory properties are influenced by many factors which, in the case of computational studies, would need to be investigated with refined techniques such as first-principles calculations. In the development of various adaptive composite materials for biological applications with embedded shape memory components, other coupled field properties of smart materials, such as piezoelectric, flexoelectric, auxetic, may also become important in the analysis and their applications at the device level [77]. At the smaller scales, shape memory effects and martensitic transformations have also been observed in superlattices [78].

In the meantime, the interest in graphene oxide and carbon-based composites with shape memory effects have been grown significantly over the last few years [1,52,79,80,81]. Among multiple promising directions, this interest also includes research studies in the context of sustainability pertinent to biomass as the associated carbon source [82] and to 4D printing technologies [83,84]. Other coupled field properties mentioned above, such as auxetic, have also been a subject of studies in graphene-based 2D materials [85]. As we mentioned earlier, the issue of microstructure evolution in SMMs has been important since the development of coupled dynamic models describing phase transformations in these materials and the methods for the solution of such models [86]. This issue remains critical for our better understanding of the dynamics of shape memory polymer composites and graphene-based structure with shape memory effects [87]. With graphene- and carbon-based composites exhibiting shape memory effects, new exciting applications continue to arise. Natural sunlight-actuated shape-memory materials with reversible shape change and self-healing abilities based on carbon nanotubes filled conductive polymer composites have recently been reported in [88]. Such composites can be fast healed under IR irradiation, and as such, these natural sunlight-responsive materials are amenable to large-scale production, providing new opportunities for the design and fabrication of sunlight-actuated smart devices and soft robotics, which can be used in biomedical applications. Bioinspired shape memory graphene properties such as tunable wettability [89] can also bring new applications in these fields, as well as graphene oxide applications for shape memory actuators implemented in micro/nanomechanical systems (MEMS/NEMS, [32]), together with new GNRs with shape memory effects. Finally, we would also mention graphene’s environmental stability and staggering transport properties [19], nonvolatile memories based on graphene [35], along with other astonishing characteristics, that can lead to sustainable applications in the future. This, combined with biocompatibility, biodegradability, and unique mechanical properties, will continue contributing to the application of SMMs in the fields of biomedicine and bioengineering.

Concurrently with this, data-driven approaches, including those based on ML, have experienced remarkable advances over recent years in many applications. Nanotechnology and materials science are among those fields where such advances have been largely due to the development of ML potentials, bridging the gap between the efficiency and accuracy in DFT and (classical) MD calculations (e.g., [90]). Depending on the ML models and descriptors used, some of the most common methodologies in this group can be differentiated by the ways we control the degree of freedoms, which can be done efficiently through the MLIP functional forms already mentioned at the beginning of this section. Among such functional forms that are used in applications, we mention Gaussian Approximation Potentials (GAP), Moment Tensor Potentials (MTP), Neural Network Potentials (NNP), Spectral Neighbor Analysis Potentials (SNAP), and their numerous modifications. Like other MLIPs, in the analysis of a structure, the MTP method encodes its local atomic environments into atomic descriptors (rotationally covariant tensors in this case), which then can be passed via an embedding function. In principle, the way to adjust the performance of the MTP, like any other MLIPs, would be to play with the number of descriptors and/or the complexity of their embedding functions [91]. However, while pushing MLIPs to their limits of the model space where we seek an efficient trade-off sampling between lower computational complexity and high resulting accuracy, problems with phase transformations have particularly serious challenges. In this paper, such problems have been addressed in the context of shape memory GRNs with the MLIP methodology for the first time (with the exception of [34], where a preliminary analysis was reported).

The MLIP methodology is moving gradually to address other complex problems, which recently also included the development of accurate interatomic potentials to study dislocation problems, point defects, and their clusters in certain materials (e.g., in bcc iron and tungsten [92]). While, to our best knowledge, no other work reported the development of MLIPs, and MTPs in particular, for the materials with phase transformations, recent attempts have been made in advancing algorithms for training MTPs on such materials as random alloys (e.g., MoNbTa medium-entropy alloys), which can be considered as a first step in simulating multicomponent systems with this methodology [93].

Our choice of MTPs in this paper was motivated by several factors. Given the recent benchmark developments for MLIPs [94], the interest in the development of MLIPs based on MTP frameworks has increased and a number of new problems have been successfully attacked, including problems with defects (not only point defects mentioned above, but also those caused, e.g., by radiation damage in bcc iron [95])). Examples of data sets that were considered include bcc (Li, Mo) and fcc (Cu, Ni) metals, as well as diamond group IV semiconductors (Si, Ge) to showcase a range of crystal structures and bonding. Such data sets were generated by using high-throughput density functional theory (DFT) calculations. With machine learning interatomic potentials (IAPs), various local environment descriptors can be used, e.g., atom-centered symmetry functions (ACSF), smooth overlaps of atomic positions (SOAP), the spectral neighbor analysis potential (SNAP) bispectrum components, as well as moment tensors. The new frontier in the development of IAPs is considered exactly that: machine learning of the quantitative relationship between local environment descriptors and the potential energy surface of a system of atoms [95]. While all descriptors mentioned above have demonstrated superior performance, compared to classical IAPs, in predicting energies, forces, and some properties, our choice in this paper has been the MTP methodology allowing a robust trade-off between accuracy and computational cost. What these works and developed benchmarks demonstrated is that the MTP can provide high accuracy at relatively low computational cost, and it is capable of accurately describing the short-range and (near) equilibrium interactions within a unified IAP model. For other applications in bioengineering discussed earlier in this section, other methods for ML potentials may prove to be useful as well, including GAP, NNP, SNAP, etc.

MLIPs can be considered as a data-driven development of approaches based on classical potentials. Such approaches are relatively easy to derive from quantum mechanics arguments via physics-based representations, leading to a lower computational cost compared to MLIPs. Consequently, although MLIPs are considered to be more robust compared to classical potentials, providing better accuracy, these features frequently come at a higher computational cost. As a result, there is a recent surge of research directed towards lowering the complexity of MLIPs and increasing their physical interpretability, while preserving their near-DFT accuracy [91]. At the same time, the quality of all data-driven approaches that are based on interpolation (and extrapolation) of the data from first-principles calculations rests on a prudent choice of training databases and the descriptors representing atomic structures. There is a wealth of atomic descriptors that have been developed over the recent decade (see, e.g., [96] and references therein), including those based on pattern learning from electronic densities of states [97] and on scaling wavelets transformation [98]. Much research has been carried out recently on the construction of physics-(quantum-mechanics)-informed ML descriptors [99], including those based on physical observables, e.g., via various approximations for calculating the self-energy on many-particle systems [100].

While in this paper, we have focused on bioengineering applications, the application of the techniques developed here could prove to be useful in other fields such as complex energy materials (mentioned earlier in Section 1), molecular and condensed systems, where the interest in MLIPs algorithms have substantially increased in recent years [101]. In addition, one should not forget that we frequently have to deal with noisy atomic structural data obtained from experiments or computations. In this latter case, probabilistic approaches capable of obtaining uncertainty estimates, such as those based on Bayesian-deep-learning models (BDLMs), can prove to be very useful [102]. Any data-driven approach of interest here, whether based on MLIPs or BDLMs, will require a mapping of the input coordinates, physico-chemical species, and other characteristics (e.g., for crystalline atomic structures, that includes the lattice periodicity) into a suitable descriptor or descriptors. As seen in Section 2, the latter can be interpreted as vectors that are invariant under rigid translations and rotations of the input structure, as well as under permutations of same-species atoms [102]. Clearly, the confidence in ML or DL models would be substantially greater if physics, implied by these invariant properties, is respected in the construction of such mappings. The MPT methodology, considered here, incorporates this physical invariance.

## 5. Conclusions

Motivated by a wide range of applications of materials with shape memory effects in biomedical engineering, which include a growing number of graphene-based structures, in this paper we have paid special attention to GNRs with shape memory effects. A pair of physics-based ML potentials (based on MTP) has been developed to relax two-dimensional GO structures, each of them targets for different energy prediction range and accuracy. Long-range interactions due to the magnetism found in ground state structures affect the ML accuracy. A third potential has been generated for energy prediction of GO nanoribbons close to ground state configurations, which allows the study of samples with thousands of atoms, computationally expensive for a purely DFT approach. The ML potential has been used to analyze the evolution of the two-phase feature for nanoribbons with different widths. The suppression process begins around ∼50nm and finishes around ∼6nm nanoribbon’s width, although the magnetization associated with the second phase is still present for 3nm nanoribbon’s width. The ML potential feature predictions for a 3nm nanoribbon’s width have been validated by DFT results.

Furthermore, the ML potential has been used to analyze different defects that can be found in realistic GO nanoribbons. We found that the two-phase shape memory behavior is destroyed for 10% of oxygen vacancy concentrations, but it is stable in front of line defects that break the high oxygen order, provided that the generated strip domains are wide enough to show the two-phase feature on their own. As such, the problems considered here involve phase transformations and, even under simplifications made, are substantially more complex and notoriously more difficult compared to those where MLIP techniques have been applied so far. Moreover, we also found that the two-phase shape memory behavior can be strongly affected by the introduction of nitrogen atoms replacing oxygen atoms, compared to the substitution of oxygen by boron atoms. We expect that future research will involve the generation of an ML potential to account for magnetic effects and the stability of larger structures at non-zero temperatures. Finally, an overview of the current and potential applications of SMMs in biomedical applications has been provided in the context of data-driven approaches, in particular physics-informed MLIP techniques.

## Figures and Tables

**Table 1:** Energies and angles for the two minima obtained with the MTP and DFT: initial calculations.

MTP $a_{\mathrm{lat}}$ (Å)	MTP Energy (meV)	MTP C-O-C Angle (deg)
15.45	101.86	102.1
18.02	101.20	130.8
**DFT $a_{\mathrm{lat}}$ (Å)**	**DFT Energy (meV)**	**DFT C-O-C Angle (deg)**
15.45	52.1	101.8
18.56	0.0	136.2

## Data Availability

The datasets that support the findings of this study, with a repository with potentials and configuration files specific to this paper, are available from the authors upon reasonable request.
